# Perceived barriers and facilitators to Risk Based Monitoring in academic-led clinical trials: a mixed methods study

**DOI:** 10.1186/s13063-017-2148-4

**Published:** 2017-09-11

**Authors:** Caroline Hurley, Carol Sinnott, Mike Clarke, Patricia Kearney, Emmy Racine, Joseph Eustace, Frances Shiely

**Affiliations:** 1 Health Research Board-Trials Methodology Research Network (HRB-TMRN), National University of Ireland, Galway, Ireland; 2 Department of Public Health and Primary Care, School of Clinical Medicine, University of Cambridge, Cambridge, UK; 3 Centre for Public Health, Queen’s University Belfast, Belfast, Ireland; 4 Department Epidemiology and Public Health, University College Cork, Cork, Ireland; 5 Health Research Board – Clinical Research Facility, University College Cork, Cork, Ireland

**Keywords:** Risk-based monitoring, ICH-GCP, Monitoring

## Abstract

**Background:**

In November 2016, the ICH published a requirement for sponsors to develop a systematic, prioritised, risk-based approach to monitoring clinical trials. This approach is more commonly known as risk-based monitoring (RBM). However, recent evidence suggests that a ‘gold standard’, validated approach to RBM does not exist and it is unclear how sponsors will introduce RBM into their organisations. A first step needed to inform the implementation of RBM is to explore academic trialists’ readiness and ability to perform RBM. The aim of this paper is to identify the attitudes and perceived barriers and facilitators to the implementation of RBM in academic-led clinical trials in Ireland.

**Methods:**

This is a mixed-methods, explanatory sequential design, with quantitative survey followed by semistructured interviews. Academic clinical researchers (*N* = 132) working in Ireland were surveyed to examine their use and perceptions of RBM. A purposive sample of survey participants (*n* = 22) were then interviewed to gain greater insight into the quantitative findings. The survey and interview data were merged to generate a list of perceived barriers and facilitators to RBM implementation, with suggestions for, and solutions to, these issues.

**Results:**

Survey response rate was 49% (132/273). Thirteen percent (*n* = 18) of responders were not familiar with the term risk-based monitoring and less than a quarter of respondents (21%, *n* = 28) had performed RBM in a clinical trial. Barriers to RBM implementation included lack of RBM knowledge/training, increased costs caused by greater IT demands, increased workload for trial staff and lack of evidence to support RBM as an effective monitoring approach. Facilitators included participants’ legal obligation to perform RBM under the new ICH-GCP guidelines, availability of RBM guidance and perception of cost savings by performing RBM in future trials.

**Conclusion:**

The results of this study demonstrate a need for training and regulatory-endorsed guidelines to support the implementation of RBM in academic-led clinical trials. The study provides valuable insights to inform interventions and strategies by policy-makers and clinical trial regulators to improve RBM uptake.

**Electronic supplementary material:**

The online version of this article (doi:10.1186/s13063-017-2148-4) contains supplementary material, which is available to authorized users.

## Background

In 1996, the International Conference on Harmonisation (ICH) published the first Good Clinical Practice guidelines (GCP) for clinical trial conduct [1]. Under ICH-GCP, sponsors in America, China and the European Union are legally obliged to monitor their trial activity [1]. Monitoring aims to protect the rights and wellbeing of trial participants, support accurate data collection and ensure compliance with regulatory requirements [1]. Traditionally, trials were monitored through intensive on-site monitoring visits with 100% source data verification (SDV) [2]. SDV can be a labourious task because it involves the validation of data presented in Case Report Forms (CRFs) against original source data, such as Consent Forms, irrespective of the trial’s risk profile [2]. Risks associated with the Investigational Medicinal Product (IMP), the vulnerability of the study population and the robustness of the study design are not considered when developing a traditional trial monitoring plan [2, 3].

In recent years the scale, complexity and costs of clinical trials have increased beyond the scope of the original ICH-GCP [4]. In November 2016, the ICH published the Integrated Addendum to ICH E6 (R1): Guideline for Good Clinical Practice E6 (R2) to respond to the changing clinical trial landscape [4]. Under the revised ICH-GCP guidelines, risk-based monitoring (RBM) is now mandatory for all trials [4]. RBM incorporates both centralised monitoring conducted off-site through an examination of data captured on an electronic data capture system (EDC) and on-site monitoring practices that are proportional to the risks associated with the clinical trial [5]. These risks relate to the Investigational Medicinal Product (IMP), the study population, research team expertise and the robustness of the study design [6, 7]. When developing a RBM plan, the trial’s protocol must be formally assessed to identify risks within the trial that can be mitigated through either on-site and/or centralised monitoring [8]. Accordingly, risk assessment is the cornerstone of RBM [9]. The emphasis on RBM is due to the assumption that it prevents waste of valuable clinical trial resources, such as study budget and staff time, on unnecessary monitoring activity that does not improve participant safety or data quality [10, 11].

The Organisation for Economic Co-operation and Development (OECD) recommends that clinical researchers to use a RBM tool when developing a RBM plan [12]. Such tools have two functions: first they support the assessment of risk in a clinical trial protocol and second they provide guidance for subsequent monitoring activity (on-site/centralised) that can mitigate the risk identified [12]. We recently published a systematic review that identified 24 RBM tools that met the OECD’s criteria [13]. However, there were many differences between the tools in terms of mode of administration (paper-based versus software as a system), the baseline risk assessment process and guidance for on-site and centralised monitoring [13]. For example, the Medical Health Regulatory Authority (MHRA) advise 100% centralised monitoring for low-risk, phase III trials, while the Swiss Clinical Trial Organisation (SCTO) advise both on-site and centralised monitoring for similar low-risk trials [5, 8].

In the absence of a ‘gold standard’ approach to RBM, it remains unclear how sponsors will implement it into their clinical trial units [14]. Given this context, it is important to establish how prepared academic trialists are to perform RBM [8]. Presently, Ireland does not have a national strategy to support the introduction of RBM into its publicly funded, academic run clinical trial units. A first step in the development of such a strategy involves the identification of academic trialists’ readiness and ability to perform RBM [14]. In this study we explore the experience of, attitudes to, and perceived barriers and facilitators associated with, the implementation of RBM in academic-led clinical trial units in Ireland.

## Methods

### Design

**Fig. 1:**
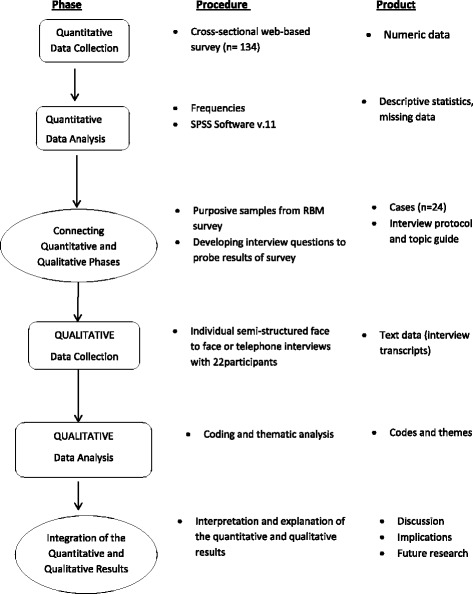
Study design – mixed-methods, explanatory sequential design procedure [15]

We used a mixed-methods, explanatory sequential design. This design occurs in two distinct but interactive phases [15]. It begins with the collection and analysis of quantitative data, followed by qualitative data collection and analysis to further explore the quantitative results (see Fig. 1) [15]. In this study, methods were combined for complementarity, where each method addressed a different aspect of the research question. The quantitative phase collected numerical data on the uptake of RBM in Ireland and its associated uses. The quantitative results facilitated sampling and development of the subsequent qualitative phase which further examined the barriers and facilitators associated with RBM [16].

### Phase 1 – Quantitative surveys

#### Survey development

The study survey was adapted from the Clinical Trials Transformation Initiative (CTTI) monitoring questionnaire [17]. The CTTI questionnaire contained 55 questions, collecting information on institutional demographics, overall study oversight methods, the use of risk- based monitoring, factors that influence monitoring risk assessments, and details on on-site and centralised monitoring practices [17].

Our survey is a shortened and modified version of the CTTI questionnaire. Questions pertaining to a trial’s governance and verifications performed during on-site monitoring visits were excluded from our survey as they were not relevant to the current study. Our study also included additional questions on RBM tools which were not explored in the CTTI survey. In total, our survey contained 20 questions. These include question regarding the participants’ demographics and their experience and understanding of the three primary components of RBM which are (1) risk assessment, (2) on-site monitoring and (3) monitoring. A number of questions focussed on respondent’s clinical trial experience since the introduction of the European Communities-Clinical Trials on Medicinal Products for Human Use Regulations to Ireland in 2004 [18]. The full survey can be found in Additional file 1.

The survey questions required responses that were either yes/no, multiple choice or open ended. Before distribution, the survey was pilot tested with a sample of 10 clinical researchers and further modification was not required.

#### Recruitment

The Health Research Board-Clinical Research Coordination Ireland (HRB-CRCI) is an independent, integrated, national clinical research network [19]. It was established in 2014 to provide centralised support in the conduct of multicentre clinical trials across Ireland [19]. Currently, the HRB-CRCI operates as a collaborative partnership with five Clinical Research Facilities/Centres (CRF/Cs) based in five universities across Ireland [19]. Researchers working in the CRF/Cs were eligible to participate in the survey. Participants included principle investigators (PIs), pharmacists, study physicians, nurses, project and quality managers, study monitors and biostatisticians.

#### Data collection

The survey was administered via Survey Monkey, an online Cloud-based survey development software [20]. Participants received the survey invitation via an email sent from the Director of the Clinical Research Facility, Cork. This email was sent to participants between February and April 2016. It contained an online link to the survey. One reminder email was sent to all, 3 to 6 weeks after the initial email. The online survey was open for 10 months from February to November 2016. However, 70% of survey responses were collected between February and June 2016.

#### Data analysis

Data captured in Survey Monkey were downloaded into Excel and then exported into SPSS version 11 for analysis.

### Phase 2 – Qualitative interviews

#### Methodology

Thematic analysis was used to identify barriers and facilitators to the implementation of RBM in participants’ past, present and future clinical trials [21].

#### Recruitment

Recruitment took place over 4 weeks from 26 September to 24 October 2016. Eligible participants were identified from respondents to the online survey who had answered survey question 8.4, 9.3 or 10.2, ‘Since 2004, have you implemented a RBM plan in a clinical trial?’ Responders of question 10 (*n* = 107) were grouped into three categories (A, B or C) based on their response; *group A* answered ‘Yes’; *group B* answered ‘No’; and *group C* answered, ‘I am not familiar with the term “risk-based monitoring”’.

#### Sampling

A purposive sample (*n* = 24) of different clinical researchers (PIs, nurses, physicians, monitors, pharmacists, managers, biostatisticians) were selected from *group A* (*n* = 8), *group B* (*n* = 8) and *group C* (*n* = 8) and invited to participate in the interviews via an email invitation. Two participants declined the invitation due to work commitments.

#### Setting

Face-to-face or telephone interviews (if the participant was not available for face-to-face) were conducted by an independent researcher (CH) from 7 October to 29 November 2016. Face-to-face interviews were conducted in a private room in each participant’s work place.

#### Data collection

A semistructured topic guide was developed to guide data collection. The topic guide was based on the results from phase 1. The topic guide included open-ended questions including: the participant’s most recent clinical trial experience including how this trial was monitored; their understanding and attitudes towards RBM; foreseen benefits and limitations of RBM; and factors that would facilitate or hinder them from implementing RBM in future trials. The topic guide was piloted on three clinical researchers based in the Clinical Research Facility, Cork and minor revisions were made. Revisions involved the inclusion of three questions pertaining to participants’ past clinical trial monitoring experience. These questions were included to gain a greater insight into the participant’s clinical trial experience. The full topic guide can be viewed in Additional file 2.

All participants received a Patient Information Leaflet and consent was obtained from all participants for their interview to be audio-taped and the content to be used for research purposes. Interviews lasted between 20 and 35 min. Data saturation was reached when additional information relating to barriers and facilitators to RBM implementation was no longer obtained from interview participants [22]. Data saturation was assessed independently for groups A, B and C. Data saturation was reached for *group A* after interview 5, *group B* after interview 4 and *group C* after interview 3. However, all scheduled interviews were conducted, transcribed and analysed.

#### Data analysis

Interviews were audio-recorded and transcribed verbatim. Transcribed interviews were coded and analysed by two coders CH (epidemiologist and clinical trial methodologist) and ER (social policy researcher) using the qualitative data analysis software NVivo [23]. The analysis followed the six phases of thematic analysis outlined by Braun and Clarke which include familiarisation with the data, generating initial codes, searching for, naming, defining and reviewing themes and producing a report [21]. The main themes were found after repeated reading of the interview transcripts, paying careful attention to barriers and facilitators associated with the implementation of RBM in past, present and future clinical trials. Barriers were defined as perceived obstacles that would prevent or impact clinical researchers’ implementation of RBM [24]. Facilitators were defined as processes that would support RBM implementation [24]. Emerging themes were organised hierarchically in three levels of analysis. At the first level are texts relating to the barriers and facilitators associated with RBM implementation that were identified across the data set. At the second level are the subthemes, where different codes were combined because they shared an underlying meaning. At the third level are the main barriers and facilitators associated with the implementation of RBM.

### Data integration

The Good Reporting of a Mixed Methods Study (GRAMMS) framework was used to inform reporting of the findings [25]. The survey and interview data were integrated at the data interpretation phase using the method of merging data [26]. Merging occurs when researchers bring two data bases together for analysis and comparison [26, 27]. In this study, the research team conducted separate analyses of the quantitative survey data and the qualitative interview data in parallel. Qualitative information was used to explore quantitative information collected in phase 1, as dictated by the explanatory sequential design [28].

### Ethics

The study received ethical approval from the Research Ethics Committee of the Cork Teaching Hospitals (CREC). Informed consent was received from all study participants.

## Results

### Participant characteristics and RBM uptake

The survey response rate was 49% (132/273). Characteristics of the survey participants are described in Table 1. Forty percent of respondents were PIs (*n* = 53). Most respondents had over 6 years’ experience in working in clinical trials (57%, *n* = 76) and over half had conducted an international multicentre trial (*n* = 93, 70%).

**Table 1:** Online survey participants’ characteristics and use of clinical trial monitoring

Variable	Total (*n* = 132)	%
Participants – clinical trial role		
Principal investigator (PI)	54	41%
Clinical trial nurse	35	26%
Project manager	21	16%
Quality manager	4	3%
Study physician	5	4%
Study monitor	5	4%
Biostatistics	2	1%
Pharmacists	6	5%
Number of participants who have conducted the following type of trial		
Industry-/commercial-led, regulated clinical trial	99	75%
Academic-led, regulated clinical trial	104	78%
Non-regulated clinical trial	79	60%
Clinical trial experience (years)		
< 1	5	4%
1–3	37	28%
4–6	14	11%
> 6	76	57%
Conducted an international multicentre clinical trial		
Yes	93	70%
No	39	30%

Survey findings showed that 37% (*n* = 49) of responders had conducted RBM since 2004. However, regardless of prior RBM experience, all survey participants said that the Investigational Medicinal Product (IMP) under investigation, the phase of the clinical trial and the experience of the study team were the main factors that they would use to determine how often a study monitor needed to visit a trial site to perform on-site monitoring. Survey responders reported that several protocol deviations or a high dropout rate would warrant additional/triggered on-site monitoring.

In total, 24 survey respondents from four of the five CRF/Cs were invited to participate in the semistructured interviews. Twenty-two interviews were conducted (RR = 92%). Interview participants included PIs (*n* = 6), nurses (*n* = 5), monitors (*n* = 3), study physicians (*n* = 3), quality managers (*n* = 3), biostatistician (*n* = 1) and trial pharmacist (*n* = 1).

### Barriers associated with the implementation of RBM

#### Lack of knowledge/training

The survey results showed that 14% (*n* = 18) of responders were not familiar with the term RBM. Of the participants who did not conduct a risk assessment in the most recent clinical trial that they worked on (*n* = 35), 17% felt that they did not have the expertise to perform a risk assessment (Table 2). Over 80% (*n* = 114) of survey responders categorised barriers to implementing centralised monitoring. Almost two thirds of these participants (62%, *n* = 71) identified lack of education as a very important barrier (Table 3). The interview data confirmed that several participants had not used RBM in past trials because they were not familiar with this type of monitoring and many did not know that RBM would be introduced in the new ICH-GCP guidelines:

**Table 2:** Reasons why survey responders did or did not conduct a risk assessment prior to developing the monitoring plan

Participants who did conduct a risk assessment (*n* = 50)	%	Participants who did not conduct a risk assessment (*n* = 35)	%
Facilitators		Barriers	
To improve patient safety	43 (86%)	Question not relevant, developing monitoring plan is a sponsor duty	15 (43%)
To improve data accuracy	32 (64%)	It is not a GCP requirement	7 (20%)
To fulfil GCP requirements	29 (58%)	Do not have the expertise to perform a risk assessment	6 (17%)
To determine a schedule for on-site monitoring visits	21 (42%)	It is too time consuming	6 (17%)
To fulfil HPRA/IMB requirements	20 (40%)	It will not improve patient safety	2 (6%)
To reduce monitoring costs	8 (16%)	It is too expensive	1 (3%)

*GCP* Good Clinical Practice, *HPRA* Health Protection Regulatory Authorities, *IMB* Irish Medicines Board

**Table 3:** Perceived problems associated with the implementation of centralised monitoring (*n* = 114)

Factor	Very important	Moderately important	Not important
Lack of education and training in centralised monitoring	71 (62%)	36 (31%)	8 (7%)
Cost associated with centralised monitoring	45 (40%)	54 (48%)	13 (12%)
Information Technology (IT) demands of centralised monitoring	53 (46%)	53 (46%)	9 (8%)
Workload associated with centralised monitoring	47 (41%)	48 (16%)	18 (16%)

‘Well, just from talking to yourself, I have to admit, prior to that I hadn’t heard about this, so I wasn’t aware that the GCP was going to be changing’ (Study physician-1).

Several interviewees, who had not conducted RBM in past trials, felt that they did not have sufficient RBM training to confidently perform RBM in future trials:

‘It would come down to the practical aspects on how is risk defined …what information are people using to make that judgement. How is it actually implemented? But ultimately I’d have to understand that before I could say I was happy to do it’ (PI-1).

They did not feel able to classify clinical trial risks and to translate these risks into monitoring activity. Similarly, some interviewees who had conducted RBM in past trials still felt ill-equipped to perform RBM in their future trials:

‘We would say we have conducted a type of risk-based monitoring, but it’s getting to the actual nitty-gritty of exactly what fields you’re going to look at and exactly what parameters are in those fields. I would say that I’d be still a bit unsure of that’ (Nurse-1).

Survey responders reported having limited experience of using centralised monitoring for essential monitoring activity such as assessing protocol compliance, inspecting informed consent and recording pharmacovigilance information. Lack of education was the main reason that survey participants did not perform centralised monitoring (Table 3). A small number of participants from the five CRF/Cs (*n* = 17) reported having a Standard Operating Procedure (SOP) for centralised monitoring in their CRF/C. However, over a third of participants (*n* = 48) were unsure if such a SOP existed in their CRF/C. Analysis of the qualitative interviews showed that some study nurses and monitors did not know how centralised monitoring could replace on-site monitoring. One participant felt that on-site monitoring offered better governance of junior clinical trial staff. This participant felt that centralised monitoring would result in monitors having less oversight of clinical trial activity:

‘As sponsor, all of the monitoring is on-site, and that’s for two reasons…, because it’s our first time working with a lot of these investigators and we’re not sure of their experience in running regulated trials, we want to make sure that they understand what’s required and what they need to do in terms of quality’ (Monitor-2).

#### Increased cost caused by greater Information Technology (IT) demands

Almost half the survey responders identified IT demands (46%, *n* = 53) and cost (40%, *n* = 45) as problems associated with the implementation of centralised monitoring in past and future clinical trials (Table 3). The interview data revealed that this perception was related to higher costs associated with EDC systems. Some interviewees felt that centralised monitoring would be costly to run as they would have to store trial data on an EDC system:

‘As sponsor, all of the monitoring is on-site…, because we don’t have electronic data capture in any of these studies because they’re not commercial studies – they’re usually grant funded, or just the PI – so there’s very little money, you’re using paper CRF’*.*(Monitor-1)*.*

This was a particular concern for trialists working on smaller trials. They felt that they would not have sufficient budget to support an EDC system and were only resourced to conduct on-site monitoring:

‘Some of the eCRFs, let’s say that company that we had, you could be talking nearly half a million, a million to get it up and running, and what small study has that if you’re talking about an oncology study which has maybe 10 patients coming into it? An eCRF is not going to be worth the set-up costs. So they’ll stick to the paper’ (Monitor-3).

#### Increased work load

Survey findings showed that perceived work load was the main reason why responders did not conduct a risk assessment prior to developing the monitoring plan for their most recent trial (Table 2). Forty-one percent of survey responders (*n* = 114) thought that increased workload was a barrier associated with the implementation of centralised monitoring (Table 3). Interviewees, who had previously conducted centralised monitoring, felt that it resulted in more administration work for trial sites as they had to support trial monitors by scanning and uploading site documents to EDC systems:

‘I noticed one of the girls downstairs was saying in the last couple of weeks… this company kept saying, “We still don’t have the CV,” and she’d sent it three times to them. So you need to have good people at the other side doing the monitoring and stuff like that. It’s just if it’s maybe stuff from the trial master file that they’re not here checking and consent forms and that. That probably might add some work’ (Nurse-3).

Some interview participants felt that sponsors would use RBM as an excuse to perform less on-site monitoring and more remote monitoring. These participants felt that a reduction in on-site visits would results in a trial monitors spending less time on site checking trial documentation such as patient Consent Forms. These participants felt that study nurses may be expected to do extra administration tasks to support trial monitors perform remote monitoring:

‘They have these centralised systems now where everything is stored centrally and it’s, like, *“*Logon and you’ll find the latest version of your protocol”. So you have to complete training for that system, you have to logon every time the new protocol is available or whatever. The onus is on the site to print it off. The onus is on the site to do everything and it’s just more and more it’s on the site, and we are not paid adequately for everything that we are being requested to do. It’s our admin staff as well. It’s like they’re just working for the pharma companies. There’s just a huge amount of resources, and it’s not accounted for’ (Nurse-4).

#### Lack of verification

The survey found that 27% (*n* = 35) of responders did not conduct a risk assessment prior to developing the monitoring plan for their most recent clinical trial. Some participants did not conduct a risk assessment because they felt that it was not a GCP requirement and would not improve patient safety (Table 2). However, these participants did use an informal process to determine what level of on-site monitoring was required for their clinical trial. Also, 21% of survey responders (*n* = 28) reported previous RBM experience.

Interview analysis showed that participants perceived a lack of scientific evidence supporting RBM and saw this as a potential barrier to its implementation in their future clinical trials. Many felt that sufficient proof did not exist to confirm that RBM was at least as effective and efficient as the 100% SDV on-site monitoring process that they currently used:

‘So it’s just our experience that the more frequent the monitoring the better. I have a negative attitude towards already a negative perception of the risk-based monitoring because 100% source data verification is what I would prefer’ (Nurse-1).

Some interviewees believed that RBM would lead to a greater reliance on centralised monitoring and a move away from on-site monitoring:

‘I know the new ICH-GCP guidelines are more into the technology, and I know that’s the way we’re going and things like that. At the end of the day, I don’t think it fully replaces the on-site’ (Nurse-2).

Many felt that the merits of centralised monitoring had yet to be proven and so were not comfortable conducting RBM in future trials if it meant fewer on-site visits:

‘I suppose the fact that things are going more electronic and it is more EDC-based. It’s the management of stuff that cannot be converted into EDC and how that’s going to be verified and how that’s going to be monitored’ (Monitor-1).

### Facilitators

#### Necessary requirement/mandate

Compliance with GCP was the main criterion participants considered when selecting a RBM tool. Of the 35 survey responders who did not conduct a risk assessment of their most recent monitoring, 20% (*n* = 7) of these participants attributed this to the absence of a GCP requirement to do so (Table 2). Correspondingly, the interview data confirmed that fulfilling GCP requirements would now motivate them to conduct RBM in future trials:

‘Yes, we will because it will be ICH-GCP will require us to do so’ (Monitor-3).

A number of interview participants said that adapting monitoring to the level of risk was a justified addition to ICH-GCP:

‘It does make sense that there’s some degree difference of risk, and therefore that the regulator environment would recognise that’ (PI-2).

They viewed the new requirements positively because they felt that RBM was a more sustainable approach to monitoring than existing approaches:

‘The landscape of clinical research has been changing, and is always changing, and just changes, changes… It is inevitable because the days of 100% source data verification is just not sustainable, really. But yeah, no, we’re definitely going to go down that route, so we are, when we get ourselves together. You know, we get more experienced, and get a bit of training’ (PI-4).

Similarly, several participants said that they would implement RBM if it became a funding or publication requirement:

‘People will put it into practice if it helps them to get funded or it helps them to publish their work’ (Biostatistician-1).

#### Availability of, and need for, guidance

Survey results suggest that more regulatory guidance would have facilitated the use of RBM in past trials (Table 2). Similarly, most interview participants believed that the introduction of regulatory-endorsed guidelines would facilitate the implementation of RBM. In Ireland, clinical trials are regulated by the Health Protection Regulatory Authorities (HPRA) and some participants suggested that this organisation should lead the way in RBM implementation:

‘I think it would be very important to have the HPRA involved, because, as you know, they come and monitor our studies’ (Monitor-3).

*‘*Well, somebody from the HPRA. There need to be quality and regulatory affairs. Managers involved and stuff like that. HPRA maybe’ (Nurse-5).

Some participants thought that RBM would result in more efficient monitoring because monitoring activity would be based solely on the risk classification of each individual trial:

‘I think it would be useful in making sure that wastage saved, that there was proper scrutiny of patients in the study. It’s a robust means of recording data, and probably having expert, external review of any adverse events’ (PI-5).

#### Economic benefits

Perceived financial benefit of RBM served as another facilitator encouraging interview participants to perform RBM in their future trials. Participants felt that RBM could reduce trial expenditure because monitoring activity would only be done as required:

‘But I think it is more cost-effective as well and I think that is an advantage’ (Quality manager-1).

Some interviewees believed that RBM would lead to a reduction in the number of on-site visits that would be performed by a monitor in each trial. These participants thought that reduced on-site monitoring visits would lead to an overall reduction in trial expenditure on monitoring:

‘So there clearly are benefits. The first one is to the extent that you’re able to replace on-site monitoring with risk-based monitoring. You have achieved a cost saving for the sponsor of the study’ (Nurse-5).

## Discussion

As far as we are aware, this is the first mixed-methods study to investigate the perceived barriers and facilitators to RBM in academic-led clinical trials. Our survey showed that over one third of respondents had previously performed RBM. The ICH-GCP Integrated Addendum will come into effect on 14 June 2017 so that that the proportion performing RBM is likely to rise in the future. However, the survey results show that currently the majority of staff in academic CRF/Cs have no experience of performing RBM. Our qualitative analysis found a lack of RBM verification as one of the main barriers preventing interview participants from performing RBM. Most interviewees said that they would feel uncomfortable conducting RBM as they believed its effectiveness had yet to be proven. Some participants felt that RBM may reduce the quality of clinical trial monitoring by offering a less intensive monitoring approach compared to traditional 100% on-site SDV. These concerns are well founded as scientific evidence confirming the effectiveness and efficiency of RBM is sparse [29]. To date, results from the OPTIMON trial are the only ones that compare RBM to traditional 100% on-site SDV monitoring. Of note, this study lacked sufficient statistical power to demonstrate non-inferiority of the RBM approach at detecting errors in the participant consent process, notification of serious adverse events (SAEs) and incorrect application of participant’s eligibility criteria [30]. Thus, data supporting the safety and effectiveness of RBM are much needed [31]. However, irrespective of this evidence gap, to be ICH-GCP-compliant clinical researchers must implement RBM in their future trials [31, 32].

Our study highlighted three additional barriers that may inhibit the introduction of RBM into academic-led clinical trial units. These included lack of RBM knowledge/training, perceived risk of increased costs caused by greater IT demands and perceived risk of increased workload for trial staff. Lack of RBM knowledge/training was identified as a major obstacle to RBM implementation among interview participants. Many felt ill-equipped to perform the initial risk assessment phase of the RBM process. This finding was reflected in the survey results, which revealed that less than one third of responders had performed a risk assessment prior to developing the monitoring plan for their most recent clinical trial. Also, the use of risk assessment among our study population was much lower than the 87% uptake recorded among American academic clinical trialists in 2011 [17]. The low uptake of risk assessment among our study participants is a cause for concern. Under the new ICH-GCP guidelines, sponsors must base their monitoring plans on the results of a risk assessment of their trial protocol [33]. Therefore, in Ireland, the knowledge gap surrounding risk assessment must be addressed if academic trialists are to become proficient RBM practitioners [31]. Interviewees believed that the availability of regulatory-endorsed guidelines would facilitate the introduction of RBM into their academic-led clinical trial organisations. To increase the use of risk assessments, Irish clinical trial regulators should develop or select an approved RBM tool at a national level [8]. A RBM tool would provide formal instruction on how to perform a risk assessment of a clinical trial protocol [12]. Clinical trial regulators in the United Kingdom and France have already developed their own RBM tools [5, 7]. Additionally, in 2014, Switzerland became the first European country to introduce a regulation adopting risk-based categorisation into their clinical trial methodology [31]. Consequently, a new article added to the Swiss Federal Constitution, provided the legal framework to regulate human research according to the risk to which participants are exposed [31]. However, it must be noted that in Switzerland the structured risk categorisation approach was not better than an ad-hoc risk assessment approach [31]. Therefore, a RBM tool should only be used to guide risk assessment and not as a one-size-fits-all approach.

Survey findings suggest that participants are not yet equipped to perform centralised monitoring. Participants had limited experience of performing essential monitoring activity, such as inspecting informed consent and protocol compliance, through centralised monitoring. This is worrying as centralised monitoring is a primary component of RBM. As outlined in the new ICH-GCP guidelines, sponsors should use centralised monitoring to complement and reduce the extent and/or frequency of on-site monitoring [4]. The perception of centralised monitoring was explored further in the qualitative phase of this study. Interview analysis showed that some participants believed that centralised monitoring would be costly to run as they would have to store trial data on an EDC system. Participants who worked on small academic trials thought that they would have insufficient budgets to support an EDC system and so they could only conduct on-site monitoring. The practical challenges associated with centralised monitoring will impact the implementation of RBM [9, 34] because centralised monitoring is a major component of RBM [9]. If researchers do not have the resources to perform centralised monitoring, then in turn they will not be able to perform RBM as their only option is to mitigate every clinical trial risk through on-site monitoring. Research is needed to develop pragmatic solutions to the challenges surrounding the use of centralised monitoring [35]. In 2016, the Food and Drug Administration (FDA) signed an Agreement with CluePoints to explore and develop a data-driven centralised monitoring approach in clinical trials [36]. CluePoints is an IT company that offers Cloud-based RBM software. Following the FDA lead, it may be useful for other countries to develop a national SOP for centralised monitoring [36]. This may involve collaboration between academic clinical researchers and computer programmers who specialise in RBM systems.

## Conclusion

The results of this study confirm the absence of, and the need for, training and the availability of regulatory-endorsed guidelines to support the implementation of RBM in academic-led clinical trials. The results of this study should be used to inform interventions and strategies by policy-makers and clinical trial regulators to improve RBM uptake.

### Strengths/limitations

To our knowledge, this is the first mixed-methods study to focus specifically on the barriers and facilitators associated with clinical researcher’s implementation of RBM in academic-led clinical trials. The triangulation of the data enabled the in-depth examination of our findings, providing a deeper understanding of the influences at work and corroborating the interpretation of the data. This approach improves the validity of the data and increases its comprehensiveness [28]. In addition our study was reported in accordance with Good Reporting of a Mixed Methods Study (GRAMMS) framework [25].

We believe that the results of this study are generalisable to the global academic clinical trial community that operates under ICH-GCP guidelines. Our study included a sample of all researchers who would typically work on a clinical trial in an academic setting. These include PIs, nurses, physicians, monitors, pharmacists, managers and biostatisticians [4]. Our diverse study population allowed for the collection of data from all types of clinical trial staff and this increased our understanding of how RBM will be implemented into a real clinical trial setting [29].

However, our study does have some limitations. The study used a mixed-methods, explanatory sequential design, with a quantitative and qualitative component. This type of study is inherently more challenging than a single-method study design as it involves the design, conduct and data integration of two different sources [37]. Achieving true integration in a mixed-methods study can be difficult [37]. To overcome this barrier, our study used the process of ‘merging’ to accurately link and analyse the quantitative and qualitative data [27]. Integration through merging of data occurs when researchers bring the two databases together for analysis and for comparison [27]. In the design phase, a plan was developed for collecting the quantitative and qualitative data that was conducive to merging the databases [27]. The quantitative survey contained a series of questions pertaining to RBM that was similar to the questions included in the semistructured interviews. The study was also cross-sectional which meant that estimates of RBM implementation could only be assessed at the present time point [38].

It should also be noted that our study was conducted before the new ICH-GCP guidelines come into effect on 14 June 2017 [32]. A longitudinal study would allow us to track RBM uptake over time and explore its impact on clinical trial conduct and monitoring outcomes. Such a study could use the quantitative results of this study as baseline data. The response rate for the survey was 49% and, therefore, responses may represent a biased sample and may not be fully representative of all academic clinical researchers working in Irish CRF/Cs. Furthermore, the response rate for the qualitative phase of our study was 92%. The high response rate may be due to sampling bias [39]. Finally, the qualitative phase of our study used two forms of data collection, face-to-face interviews and telephone interviews. We are confident that this did not impact the qualitative findings as there were no differences apparent in the data generated by both collection methods. This observation is in line with other research which found no significant differences in the data generated by face-to-face and telephone interviews [40].

## Additional files

Additional file 1: Full monitoring survey. (DOCX 744 kb)

Additional file 2: Interview Topic Guide. (PDF 342 kb)
