# Supplementary material for: Perceived barriers and facilitators to Risk Based Monitoring in academic-led clinical trials: a mixed methods study
Source: Trials. 2017 Sep 11;18:423. doi: 10.1186/s13063-017-2148-4 (PMC5594426; doi:10.1186/s13063-017-2148-4)
Supplement: Supplementary file 1 — Full monitoring survey. (DOCX 744 kb) [file 13063_2017_2148_MOESM1_ESM.docx]

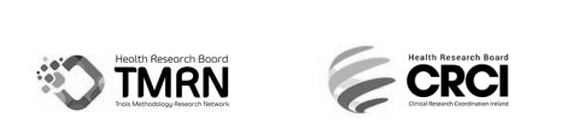


**Monitoring Survey**

**Welcome to My Survey**

# Clinical trial monitoring is mandatory under ICH-GCP guidelines. In June 2015, the ICH published the Integrated Addendum to ICH GCP, which recommends systematic, prioritised, risk-based monitoring for clinical trials.

**The aim of this survey is:**

- **To identify how academic led clinical trials are monitored in Ireland.**
- **To better understand the reasons for using the methods identified.**
- **To identify any limitations of these methods.**
- 1. Please indicate if you consent for your answers to be used for research purposes

Yes No


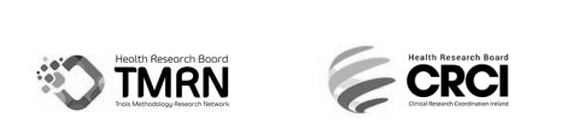


**Monitoring Survey**

# Please enter your contact details below. This information will be used to track the survey's response rate

- 2. Name :
- 3 Email address:


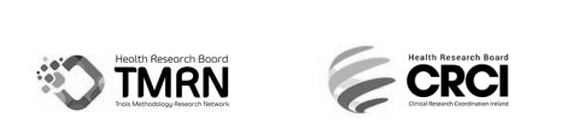


**Monitoring Survey**

**Demographics**

4.1 Please indicate the University/Clinical trial unit you are affiliated with?

- University College Cork (UCC)
- National University of Ireland Galway (NUIG)
- Wellcome Trust -HRB Clinical Research Facility at St. James Hospital
- Royal College of Surgeons Ireland (RCSI)
- University College Dublin (UCD)
- 4.2 What is your role within a clinical trial?
- Principal Investigator (PI)
- Clinical trial nurse
- Project Manager
- Quality Manager
- Study doctor
- Study Monitor
- Biostatistics
- Pharmacists

Other (please specify)

- 4.3 Please identify the Medical Therapeutic area/areas in which you work? (i.e. Neurology, Oncology, Geriatrics)
  1. How many years have you been working in clinical trial research?
- <1
- 1-3
- 4-6
- >6

4.5 Please list any specific clinical trial training that you have received? (I.e. GCP training, postgraduate training)

4.6 Since the introduction of the European Communities (Clinical Trials on Medicinal Products for Human Use) Regulation in 2004, have you conducted international multi-site trials?

Yes No

4.7 Since the introduction of the European Communities (Clinical Trials on Medicinal Products for Human Use) Regulation in 2004, how many of the following types of clinical trials have you conducted?** Regulated trials need HPRA approval


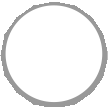

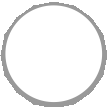

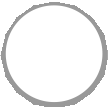

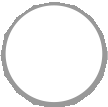
0 1 2-3 > 3


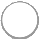

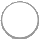

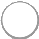

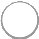


Industry/commercial-led, regulated clinical trial

Academic-led, regulated clinical trial


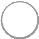

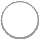

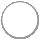

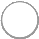


Non-regulated clinical trials

- 4.8 Since 2004, have you participated in a HPRA/ IMB clinical trial inspection?

Yes No


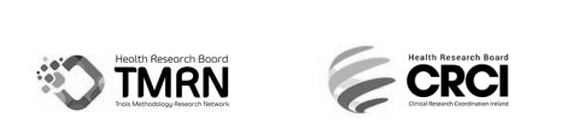


**Monitoring Survey**

**On-site Monitoring**

- 5.1 Do you think the following features of a clinical trial are important to consider when deciding the frequency of on-site visits required to monitor the trial?

Very important

Moderately

important Not important


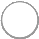

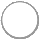

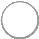


Investigational Medicinal Product (IMP)


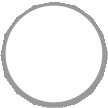

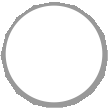

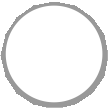
Budget
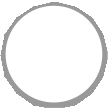

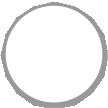

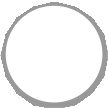


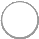

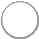

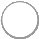


Study population

Phase of trial (I, II, III, and IV)


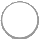

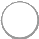

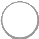


Experience of clinical trial team

- 5.2 Which of the following factors would you use to trigger an onsite monitoring visit? (Select all that apply)?

Several protocol deviations Incidence of adverse events Upcoming regulatory inspection Low recruitment rate

High subject drop-out rate Inexperience of clinical trial site other (please specify)


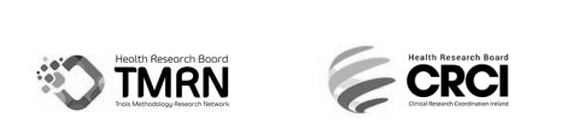


**Monitoring Survey**

**Centralised Monitoring**

# **Centralised monitoring is also known as remote monitoring. This monitoring system allows clinical researcher to remotely monitor clinical trial activity such as recruitment trends, data entry etc.

- 6.1 Does your clinical research unit have a SOP for centralized monitoring?


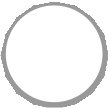
 Yes
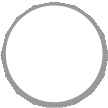
 No


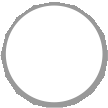
 Not sure

- 6.2 Since 2004, have you used centralised data monitoring for the following quality management activities in a clinical trial?

Yes No Not sure


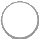

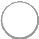

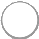


To assess protocol compliance

To record pharmacovigilance information (I.e. adverse events, SAEs)
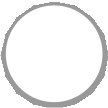

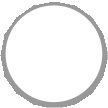

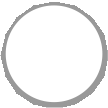


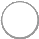

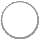

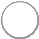


To organise sampling and material logistics (e.g. specimen collection, storage and shipment)

To guide, target or supplement on-site monitoring visits
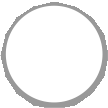

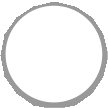

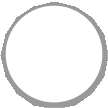


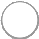

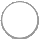

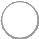


To completely replace on-site monitoring

To supervise study recruitment
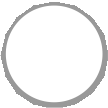

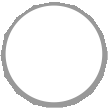

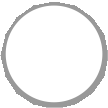


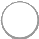

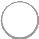

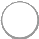


To inspect the informed consent process


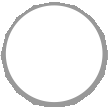

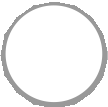

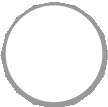
To identify missing data

- 6.3 Please indicate how important you consider the following factors to be as a barrier to implementing centralised monitoring in clinical trials?

Very important

Moderately

important Not important


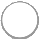

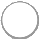

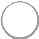


Lack of education and training in centralised monitoring

Cost associated with centralised monitoring
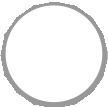

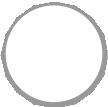

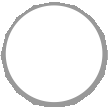


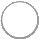

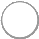

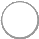


IT demands of centralised monitoring

Workload associated with centralised monitoring
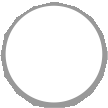

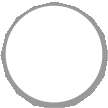

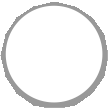


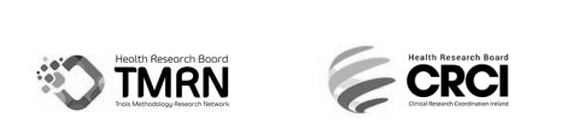


**Monitoring Survey**

**Risk-based Monitoring- for academic led clinical trials**

- 7.1 Please name the most recent clinical trial you worked on?
- 7.2 In this clinical trial did you or your study team complete an assessment of risk prior to developing the monitoring plan?


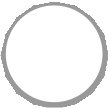
 Yes
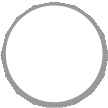
 No

if **Yes** please **only** answer questions **8.1-8.4**

if **No** please **only** answer questions **9.1-9.3**

if **Not sure** please **only** answer questions **10.1-10.2**


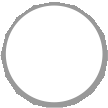
 Not sure


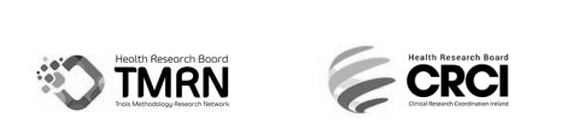


**Monitoring Survey**

**Risk-based monitoring- for academic led clinical trials**

Note: These questions are only relevant to participants that answered ‘**Yes’** to question 7.2 In this clinical trial did you or your study team complete an assessment of risk prior to developing the monitoring plan?

- 8.1 Did you or your study team use a risk assessment tool when assessing the risks in your clinical trial? **a risk assessment tool is used to identify the risks within an approved clinical trial protocol that can be mitigated through monitoring. For example a risk assessment tool could be an in-house SOP, a checklist or a computer programme**


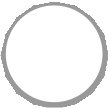
 Yes
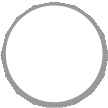
 No


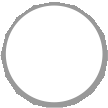
 Not sure

- 8.2 Please indicate why you or your study team performed a risk assessment? (Select all that apply)

To fulfil HPRA/IMB requirements To improve patient safety

To improve data accuracy To reduce monitoring costs

To determine a schedule for on-site monitoring visits To fulfil GCP requirements

Not sure

Other (please specify)

- 8.3 Do you think the following are important features of a risk-assessment tool?

Moderately

Very important important Not important


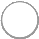

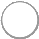

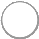


It is GCP compliant

Training is required to operate the tool
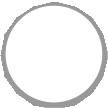

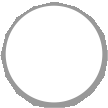

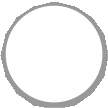


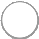

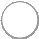

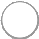


It is approved by the HPRA

It requires specialised software to operate
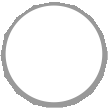

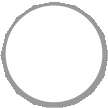

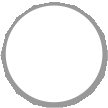


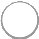

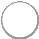

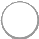


It is paper-based

It contains less than 20 risk assessment questions
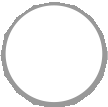

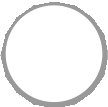

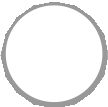


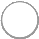

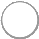

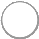


It has been formally validated for precision

It clearly defines risk and appropriate monitoring guidelines

- 8.4 Since 2004, have you implemented a risk-based monitoring plan in a clinical trial?

Yes No

I am not familiar with the term ‘Risk-based monitoring’

**Monitoring Survey**

**Risk-based monitoring - for academic led clinical trials**

Note: These questions are only relevant to participants that answered ‘**No’** to question 7.2 –

‘*In this clinical trial did you or your study team complete an assessment of risk prior to developing the monitoring plan?*’

- 9.1 Please indicate why you or your study team did not perform a risk assessment? (Select all that apply)

It is not a GCP requirement

Do not have the expertise to perform a risk assessment It is too time consuming

It is too expensive

It will not improve patient safety Not sure

Other (please specify)

- 9.2 Do you think the following are important features of a risk-assessment tool? **a risk assessment tool is used to identify the risks within an approved clinical trial protocol that can be mitigated through monitoring. For example a risk assessment tool could be an in-house SOP, a checklist or a computer programme**

Very important

Moderately

important Not important

It is GCP compliant

Training is required to operate the tool

It is approved by the HPRA

It requires specialised software to operate

It is paper-based

It contains less than 20 risk assessment questions

It has been formally validated for precision

It clearly defines risk and appropriate monitoring guidelines

- 9.3 Since 2004, have you implemented a risk-based monitoring plan in a clinical trial?

Yes No

I am not familiar with the term ‘Risk-based monitoring’

**Monitoring Survey**

Risk based monitoring - for academic led clinical trials

Note: These questions are only relevant to participants that answered ‘**Not sure’** to question 7.2 –

‘*In this clinical trial did you or your study team complete an assessment of risk prior to developing the monitoring plan?*’

- 10.1 Do you think the following are important features of a risk-assessment tool? **a risk assessment tool is used to identify the risks within an approved clinical trial protocol that can be mitigated through monitoring. For example a risk assessment tool could be an in-house SOP, a checklist or a computer programme**

Very important

Moderately

important Not important

It is GCP compliant

Training is required to operate the tool

It is approved by the HPRA

It requires specialised software to operate

It is paper-based

It contains less than 20 risk assessment questions

It has been formally validated for precision

It clearly defines risk and appropriate monitoring guidelines

- 10.2 Since 2004, have you implemented a risk-based monitoring plan in a clinical trial?

Yes No

I am not familiar with the term ‘Risk-based monitoring’

**Monitoring Survey**

# Thank You for participating in the Monitoring Survey
