# Supplementary material for: Perceived barriers and facilitators to Risk Based Monitoring in academic-led clinical trials: a mixed methods study
Source: Trials. 2017 Sep 11;18:423. doi: 10.1186/s13063-017-2148-4 (PMC5594426; doi:10.1186/s13063-017-2148-4)
Supplement: Supplementary file 2 — Interview Topic Guide. (PDF 342 kb) [file 13063_2017_2148_MOESM2_ESM.pdf]

Study: Perceived barriers and facilitators to Risk Based Monitoring in academic-led clinical trials: a mixed methods study

Document: Interview Topic Guide

Hi (participant's name) , thank you for completing the Monitoring Survey. The questions I will ask you today will further explore your view and opinions on risk based monitoring. There are no right answers so please stop me if you want me to clarify any question.

---

1. To start could you tell me about the most recent clinical trial you worked on and your role in that trial?
2. At what stage is that trial- recruitment, close out?
3. How is/was that trial monitored?
4. Did you/team use on-site and/or centralised monitoring? Did you use an electronic clinical report form eCRF
5. Who developed the initial monitoring plan? *(You, monitor, PI)*
6. Were you involved in the development of the monitoring plan?
7. Why did you/they choose this type of monitoring? *(Cost, IT, staff)*
8. Was the monitoring plan reviewed and changes during the trial duration?
9. In November 2016, the ICH-GCP will launch the updated version of GCP which will recommend clinical trial Sponsors use risk based monitoring. Are you familiar with the new guidelines
10. If no we can review paragraph from 'participant sheet'?

11. Are you familiar with the term risk-based monitoring?

12. Have you ever conducted risk-based monitoring in a clinical trial?

13. Do you think risk based monitoring will change you monitor a clinical trial in the future?

14. Do you feel there are benefits associated with RBM?

15. Do you think there are limitations associated with RBM?

16. In future trials you work on, would you consider using risk-based monitoring?

17. In these trials, would you like to be involved in the development of a monitoring plan?

18. Do you feel you have the skills and knowledge base to conduct RBM?

19. What intervention or support would help you conduct risk based monitoring?

20. Who should lead this intervention?

21. How much commitment would you give to the intervention?

22. Do you have any additional information you would like to add?

23. Do you have any questions for me?
